# Breast in vivo dosimetry by EPID

**DOI:** 10.1120/jacmp.v11i4.3275

**Published:** 2010-09-02

**Authors:** Andrea Fidanzio, Francesca Greco, Alessandra Mameli, Luigi Azario, Mario Balducci, Maria Antonietta Gambacorta, Vincenzo Frascino, Savino Cilla, Domenico Sabatino, Angelo Piermattei

**Affiliations:** ^1^ U.O. di Fisica Sanitaria Policlinico Gemelli UCSC Roma Italy; ^2^ Istituto di Fisica Università Cattolica del S. Cuore UCSC Roma Italy; ^3^ U.O. di Radioterapia Policlinico Gemelli UCSC Roma Italy; ^4^ U.O. di Fisica Sanitaria Centro di Ricerca ad Alta Tecnologia nelle Scienze Biomediche dell'Università Cattolica S. Cuore Campobasso Italy

**Keywords:** in vivo dosimetry, transit dosimetry, quality assurance in breast radiotherapy

## Abstract

An electronic portal imaging device (EPID) is an effective detector for *in vivo* transit dosimetry. In fact, it supplies two‐dimensional information, does not require special efforts to be used during patient treatment, and can supply data in real time. In the present paper, a new procedure has been proposed to improve the EPID *in vivo* dosimetry accuracy by taking into account the patient setup variations. The procedure was applied to the breast tangential irradiation for the reconstruction of the dose at the breast midpoint, $D_{m}$. In particular, the patient setup variations were accounted for by comparing EPID images versus digitally reconstructed radiographies. In this manner, EPID transit signals were obtained corresponding to the geometrical projections of the breast midpoint on the EPID for each therapy session. At the end, the ratios R between $D_{m}$ and the doses computed by the treatment planning system (TPS) at breast midpoints, $D_{m,\text{TPS}}$, were determined for 800 therapy sessions of 20 patients. Taking into account the method uncertainty, tolerance levels equal to ±5% have been determined for the ratio R.

The improvement of *in vivo* dosimetry results obtained (taking into account patient misalignment) has been pointed out comparing the R values obtained with and without considering patient setup variations. In particular, when patient misalignments were taken into account, the R values were within ± 5% for 93% of the checks; when patient setup variations were not taken into account, the R values were within ± 5% in 72% of the checks. This last result points out that the transit dosimetry method overestimates the dose discrepancies if patient setup variations are not taken into account for dose reconstruction. In this case, larger tolerance levels have to be adopted as a trade‐off between workload and ability to detect errors, with the drawback being that some errors (such as the ones in TPS implementation or in beam calibration) cannot be detected, limiting the *in vivo* dosimetry efficacy.

The paper also reports preliminary results about the possibility of reconstructing a dose profile perpendicular to the beam central axis reaching from the apex to the lung and passing through the middle point of the breast by an algorithm, similar to the one used for dose reconstruction at breast midpoint. In particular, the results have shown an accuracy within ± 3% for the dose profile reconstructed in the breast (excluding the interface regions) and an underestimation of the lung dose.

PACS numbers: 87.55.Qr, 87.55.km, 87.53.Bn

## I. INTRODUCTION

Based on the steepness of dose‐response relationships, both for local tumor control and for normal tissue complications, an accuracy requirement of 3.5% or one standard deviation (1 SD) in dose delivery in radiotherapy daily clinical practice has been formulated.^(^ ^1^ ^)^ However, systematic errors in dose delivery for an individual patient can arise, due to: (i) incorrect linac calibration, machine output and field flatness, use of beam modification devices, (ii) incorrect treatment planning system (TPS) calculations, and/or (iii) incorrect patient setup and internal organ motion.^(^ ^2^ ^–^ ^5^ ^)^ Therefore, several international organizations recommend performing *in vivo* dose measurements.^(^ ^6^ ^,^ ^7^ ^)^

Currently, the most diffused *in vivo* dosimetry method is based on the use of two diodes positioned at the beam central axis entrance and exit, respectively, on patient skin surface. Thus the patient midpoint dose, $D_{m}$, along the beam axis can be determined by a simple relationship and readings of calibrated diodes. However, this method requires: (i) periodic diode recalibrations; (ii) accurate positioning of the detectors on the patient for every gantry angle; (iii) corrections for photon fluence perturbation; (iv) corrections for temperature, angle of beam incidence and beam energy. Moreover, this method has some limitations when a patient presents asymmetric inhomogeneities along the beam central axis.^(^ ^7^ ^,^ ^8^ ^)^

In recent years, many researchers have worked on *in vivo* dosimetry examining new methods and detectors. In particular, the electronic portal imaging device (EPID) is very attractive because it supplies two‐dimensional information, does not require special efforts to be used during the treatment, and can supply, together with CT data, 3D dose reconstructions in treatments where the tissue inhomogeneities can be neglected.^(^ ^9^ ^)^ Pasma et al.,^(^ ^10^ ^)^ Boellaard et al.^(^ ^11^ ^)^ and the authors^(^ ^12^ ^)^ have proposed methods of transit dosimetry by EPID, for *in vivo* determination of dose to the patient in a reference point along the beam central axis. In particular, the method proposed by the authors is based on correlation functions defined as the ratio between the EPID transit signal and the dose to water measured in solid water phantoms.

The authors^(^ ^13^ ^)^ implemented the method previously proposed^(^ ^12^ ^)^ for *in vivo* dosimetry of breast tangential irradiation. In that work, the transit signal, $S_{t}$, along the beam central axis was measured by an ion chamber (because the EPID was an old model not suitable for dosimetry), and was correlated to the dose measured in cylindrical water phantoms to obtain the correlation functions used to reconstruct the *in vivo* patient dose at the breast midpoint, P.

In the present paper, the calibration procedure used by the authors^(^ ^13^ ^)^ for the ion chamber has been applied to obtain *in vivo* dosimetry by two EPIDs. Moreover the influence of the patient setup variations on *in vivo* dose reconstruction has been analyzed using the same EPID portal images. In order to explain this last point, let us consider that in the clinical practice, a correlation function value has to be determined for each field, dependent on the patient radiological thickness, w‘, evaluated along the beam central axis on the CT slice containing the isocenter. However, if during the beam delivery the patient setup differs from that realized for the CT scan, the radiological thickness used to determine the correlation function value does not correspond to the one crossed by beam axis during the therapy session. Since, in general, the breast shows a large curvature, even setup variations of 3–4 mm can produce, for some patients, significant variations of the radiological thickness crossed by the beam axis and, consequently, of the measured transit signal. In such cases, reconstructed dose at the breast midpoint, P, (which is no more positioned along the beam central axis because of misalignment), cannot be accurately evaluated by means of EPID transit signal measured on the beam axis. This problem can be addressed following two approaches. The first one accounts for the reduced accuracy of dose reconstruction using larger tolerance levels. In the second, breast midpoint misalignment with respect to the beam axis is determined on EPID images and the transit signal reading in correspondence of the midpoint, P, projection on the EPID is thus used. In this manner, the w’ value obtained by the CT slice can still be considered representative of the radiological thickness crossed by the beam rays which pass through the point, P. The first approach is simpler and requires less workload, but some errors would not be noticed due to the large tolerance levels. The second approach requires a major workload, but allows for smaller tolerance levels and, therefore, better detection of eventual errors. In the present paper, the implementation of a procedure to apply the second approach is proposed.

## II. MATERIALS AND METHODS

### A. The aSi‐based EPID

In this study, two aSi‐based EPIDs (aS500, Varian Medical System Milpitas, CA, USA) mounted on two linacs Clinac 2100 C/D (Varian) were used. The two linacs operating in our radiation therapy department are equipped with 120 multileaf collimators used to conform the dose to the target volume.

The dosimetric module of the Varian Vision software, version 7.3.10 SP3, implemented by the vendor for IMRT pretreatment verifications was used to acquire and calibrate the EPID images. In particular, the EPID reading on the beam axis has been calibrated to yield 1 Calibrated Unit (CU) for a $10\times 10{\text{cm}}^{2}$ field with a source to EPID distance (SED) equal to 100 cm supplying 100 Monitor Unit (MU). The EPID response reproducibility and linearity have been well‐reported in literature.^(^ ^12^ ^,^ ^14^ ^)^ In particular, reproducibility of the EPID signal was estimated to be better than ± 0.5% two standard deviations (2 SD) for different gantry angles, while the EPID signal linearity with the MUs was within ± 1% (2 SD) for irradiations with more than 50 MU.

### B. Method implementation

**Figure 1:**
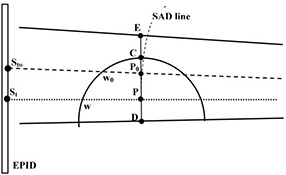
Experimental set‐up used to determine the correlation function F(w) and the dose profile along the segment $\bar{CD}$. The cylindrical phantom section shown contains the mid‐point P of the chord w positioned at the SAD along the beam's central axis (dotted line) and the point $P_{0}$ at the intersection between the segment $\bar{CD}$ and the chord $w_{0}$. The transit signals $S_{t}$ and $S_{\text{to}}$ were determined at the geometrical intersection between beam axis and the fan line (dashed line) and the EPID respectively. The continuous lines represent the geometrical field edges.

The *in vivo* dosimetry method applied here is based on correlation functions defined as the ratio between the EPID transit signals, $S_{t}$ [CU], and the midplane doses, $D_{m}$, measured in four cylindrical water phantoms (with radii, r, equal to 6 cm, 8.25 cm, 10.7 cm and 14.5 cm, respectively) irradiated on their lateral surfaces with the gantry at 90° (see Fig. 1), as specified in a previous paper.^(^ ^13^ ^)^

Dosimetric measurements were performed using 6 MV X‐ray beams modified by 15° and 30° hard wedge filters.

A PTW ion chamber model M31010 (PTW Freiburg, Germany) was positioned along the beam central axis in the midpoint, P, of different chords of the cylindrical water phantoms in order to determine the $D_{m}$ values at the source to axis distance (SAD) (Fig. 1). In particular, the chords were selected to have the ratio between the chord length, w, and the segment $\bar{CD}$ (Fig. 1), in the range between 1.5 and 5.0 cm while the length of the segment $\bar{CE}$ was fixed at 3 cm. The length of the field along the cylindrical phantom axis was varied between 16 and 24 cm. These constraints are the same ones used by the authors^(^ ^13^ ^)^ and account for the patient irradiation modality adopted by our center. The EPID was positioned at SED=150 cm, and the $S_{t}$ signals were determined as the average value of the 25 EPID central pixels. During the $S_{t}$ measurements, the ion chamber was removed from the phantom, to avoid measurement perturbation. Moreover, $S\prime_{t}$ measurements (the transit signal obtained for a generic position of the cylindrical phantom) were performed varying the distance, d, between P and the isocenter within ± 4 cm. This way the variation of the scatter contribution as a function of the phantom position was evaluated.

The $S_{t}/D_{m}$ ratios were found to be independent of beam dimensions, the wedge angle and the phantoms radius, r, within an experimental uncertainty of ± 0.5%. Therefore, the ratios $S_{t}/D_{m}$, obtained for the two wedge filters, were fitted with a polynomial of the second order, F(w), as a function of w:

(1) $$F(w)=a_{1}w^{2}+a_{2}w+a_{3}$$

where $a_{1},\;a_{2}$ and $a_{3}$ are fitting parameters.

According to previous papers,^(^ ^12^ ^,^ ^13^ ^)^ the dose, $D_{m}$, at the phantom midpoint, P, (Fig. 1) along the beam central axis can be obtained by applying the equation:

(2) $$D_{m}=\frac{S_{\;t}^{\prime }\cdot f(d,r)}{F(w)\cdot {\left(\frac{\text{SAD}+d}{\text{SAD}}\right)}^{2}}$$

where $S\prime_{t}$ is the transit signal obtained for a generic position of the cylindrical phantom, *d* is the distance between P and the isocenter (in particular d > 0 when the distance between P and the source is greater than 100 cm and d < 0 when the same distance is less than 100 cm), *f(d,r)* is an empirical factor that accounts for scatter contribution variations on the EPID as a function of the distance, d, and the cylinder radius, r. In particular, *f(d,r)* is defined as the ratio between the transit signal, $S_{t}$, obtained for d=0 and the transit signal, $S\prime_{t}$, obtained for d values ranging between ± 4 cm and $r=(w^{2}+4{\bar{\text{CP}}}^{2})/8\bar{\text{CP}}$.^(^ ^13^ ^)^

### C. Off‐axis dose reconstruction

The applicability of Eq. (2) was investigated also in relation to off‐axis positions. In particular, using the same irradiation configurations used to determine the correlation functions F(w), dose values $D_{\text{Po}}$ in points along the segment $\bar{CD}$ (e.g., the point $P_{0}$ shown in Fig. 1) were measured by the ion chamber in order to obtain a dose profile. The respective transit signals, $S_{\text{to}}$, were determined as the average signal of the 25 pixels centered on the geometrical intersection between the EPID and the fan line, passing trough the points $P_{0}$ as, shown in Fig. 1. The $S_{\text{to}}/D_{\text{PO}}$ ratios, determined for the four cylindrical phantoms along different fan lines as a function of chord length $w_{o}$ (Fig. 1), were found to agree within ± 0.5% with the F($w_{o}$) values computed using Eq. (1). This result is due to the small beam divergence (i.e., the fan lines can be considered as being almost parallel to the beam axis). This means that $P_{o}$ is very close to the midpoint of the chord $w_{o}$ and is at ~100 cm from the source. Therefore when P is coincident with the isocenter, the dose in off‐axis positions, $D_{\text{Po}}$, can be determined by:

(3) $$D_{\text{Po}}=\frac{S_{\text{to}}}{F(w_{o})}$$

**Figure 2:**
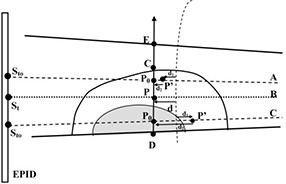
Scheme for dose reconstruction at $P_{0}$ points situated inside water and inside a low density tissue (shadowed area). P is the mid‐point on the chord along the beam's central axis B (dotted line). The P' points are at the half radiological thickness along fan lines A and C (dashed lines). The $P_{0}$ points represent the intersection between fan lines A and C and the segment $\bar{CD}$; d is the distance between P and the SAD line; $d_{1}$ is the distance between P' and the SAD line; and $d_{2}$ is the distance between P' and $P_{0}$. The transit signals $S_{t}$ and $S_{\text{to}}$ were determined at the geometrical intersection between beam axis and the fan line (dashed line) and the EPID respectively. The continuous lines represent the contours of the geometrical field.

Since the same correlation function can be used to reconstruct the dose in axis and off‐axis positions, Eq. (2) can be used to reconstruct the dose at breast midpoint, P, also when a patient misalignment has occurred. Moreover, in order to explore the potentiality of the *in vivo* dosimetry method, an expression was derived to reconstruct the dose profile perpendicular to the beam central axis and reaching from the apex to the lung passing through the middle point of the breast. For example, Fig. 2 shows a breast‐shaped phantom with the intent to explain dose profile determination along segment $\bar{CD}$ in the case d≠0. The dose, D, to a point $P_{0}$ along $\bar{CD}$ (inside the water or inside a low density material simulating the lung) can be determined as follows:

(4) $$D=\left[\frac{S_{\text{to}}}{F(w^{\prime })}\frac{f(d,r)}{{\left(\frac{\text{SAD}+d_{1}}{\text{SAD}}\right)}^{2}}\right]\cdot \left[{\left(\frac{\text{SAD}+d_{1}}{\text{SAD}+d_{1}+d_{2}}\right)}^{2}\exp(‐\frac{\mu_{\text{en}}}{\rho }w_{2}^{\prime })\right]$$

where

*F(w‘)* is the correlation function defined in Eq. (1) and evaluated for the chord radiological thickness, *w‘*;

*f(d,r)* is the empirical scatter correction factor determined for the chord coincident with the beam central axis and assumed constant for all dose points along $\bar{CD}$;

$d_{1}$ is the geometrical distance between the point at half chord thickness, *P‘*, and the SAD line ($d_{1}>0$ when P’ is at a distance from the source greater than the SAD, otherwise $d_{1}<0$);

$d_{2}$ is the geometrical distance between P' and $P_{0}\;(d_{2}>0$ when P' is at a distance from the source greater than $P_{0}$ otherwise $d_{2}>0$); *d* is the geometrical distance between P and the SAD line that, disregarding the fan line divergence, is equal to $d_{1}+d_{2}$;

$\mu_{en}/\rho$ is the water mass energy absorption coefficient equal to $2.73\times 10^{‐2}\;{\text{cm}}^{2}g^{-1}$,^(^ ^15^ ^)^ relative to a mean photon energy of 1.7 MeV for the 6 MV photon beam of a Varian linac;^(^ ^16^ ^)^

$w\prime_{2}$ is the radiological thickness calculated along the distance $d_{2}$ having the same sign as $d_{2}$.

Equation (4) was tested reconstructing off‐axis dose values in the cylindrical water phantoms. In particular, the four cylindrical phantoms were irradiated using the same geometrical configurations used to determine the f(d,r) factors (where the distance, d, was varied between +4 cm and −4 cm). The PTW ion chamber was positioned at different points (at a distance of 1 cm from each other) along the segment $\bar{CD}$ inside the water phantoms to determine the dose values along the profiles. These dose values were compared with those reconstructed at the same points for Eq. (4).

Equation (4) may be rewritten as:

(5) $$D=S_{\text{to}}\;\;\;C$$

where C is the dose reconstruction factor characteristic of each fan line.

The aim of the present work is to evaluate the *in vivo* reconstructed dose at the breast midpoint, taking into account the patient misalignment. Therefore the *in vivo* doses, $D_{m}$, at the breast midpoint have been determined for all patients, by aligning the digital portal images (DPIs) versus the digitally reconstructed radiographies (DRRs). Thus the ratios $R=D_{m}/D_{m,\text{TPS}}$ between $D_{m}$ and the dose computed by the TPS in the same points, $D_{m,\text{TPS}}$, were determined for all the checked therapy sessions. The *in vivo* reconstructed dose profiles were obtained only for one patient to realize a preliminary test of the accuracy of the method along the whole profile.

**Figure 3:**
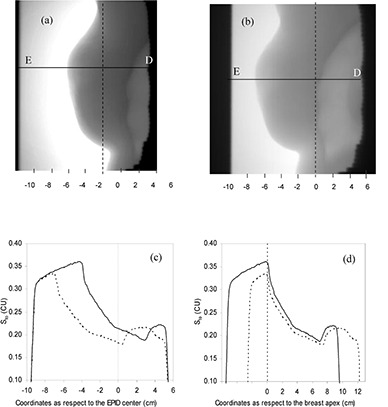
Portal image of the medial beam acquired for patient #7 with correct set‐up (a); portal image of the medial beam acquired when the patent's misalignment was 2 cm (b). $S_{\text{to}}$ profiles (c) obtained along the ED line of image 3(a) (continuous line) and of image 3(b) (dashed line); (d) the same $S_{\text{to}}$ profiles of aligned with respect to the DRR.

With the aim of describing the procedure used for the breast midpoint or profile dose reconstruction, an example of two therapy sessions obtained for a patient is reported here. C factors were determined for different fan lines by using the patient CT slice containing the isocenter. The dose profile reconstructions were performed taking into account the patient setup variations. In particular, DPIs were superimposed to DRRs of the beam views in order to obtain the coincidence of the breast profiles visible in the two images. Then a transit signal profile was read on the segment of DPI that passed through the beam axis marked on the correspondent DRR. For example, Figs. 3(a) and 3(b) show two DPIs acquired when patient setup was in agreement with the DRR (within ± 2 mm) and when a misalignment, s, of 2 cm along the direction $\bar{ED}$ was observed, respectively. Figure 3(c) shows the $S_{t}$ profiles obtained along the segments $\bar{ED}$ of Fig. 3(a) and 3(b). Figure 3(d) shows the same profiles as in Fig. 3(c), aligned on the basis of the DRR breast profile. The accuracy of the alignment process was estimated within ± 2 mm (2 SD), considering the difficulty to reproduce the same patient setup as in the CT scan without immobilization devices. Considering the breast curvatures investigated in the present paper, the ± 2 mm uncertainty of breast alignment corresponded to an uncertainty of 2% (2 SD) of the transit signal used to reconstruct the dose in the breast midpoint, P.

### D. Planning and treatment modality

The dosimetries of 20 patients treated after conservative surgery (lumpectomy or quadrantectomy) have been examined here. All treatment plans were obtained using the Anisotropic Analytical Algorithm (AAA) dose‐calculation model (version 7.5.22.0) implemented on the Eclipse TPS. The accuracy of the AAA with respect to Monte Carlo calculation in breast treatment is within 3% (2 SD)^(^ ^17^ ^)^ while, for the interface regions, deviations up to 10% (2 SD) were observed.^(^ ^18^ ^)^ The irradiations involved breast, chest wall and regional lymph nodes, with two opposite 6 MV tangential photon beams filtered using a 15° or a 30° hard wedge. Field lateral dimensions exceeded the breast apex by about 3 cm. The prescribed total dose was 50.4 Gy, delivered in five fractions per week with a daily dose of 1.8 Gy.

Patients were positioned in the supine position on a tilted plane with one hand above the head clasping the handlebar of the extended wing board (CIVCO, Kalona, Iowa, USA). Verification portal images (VPIs) were performed at the beginning of the treatment and after administration of about 10 and 20 Gy.

DPIs of the two tangential fields were acquired for 20 fractions for each patient. A total of 800 images were examined. For every patient, the R ratios over the 40 tangential fields examined have been determined. Analyzing DPIs, the average standard deviations, σ, of patient setup misalignments have been determined for the breast apex‐central lung and the cranio‐caudal directions.

### E. Tolerance levels

The tolerance level of the dose reconstructions at the breast midpoint has been analyzed, taking into account the uncertainty sources in terms of 2 SD. In particular, the levels of the principal uncertainties associated to the clinical use of Eq. (2) are:

(i) ± 1.1% for $S\prime_{i}$(w,L) due to the signal reproducibility and linearity with the monitor unit;
(ii) ± 2.0% for F(w) due to the accuracy of the fits obtained by Eq. (1) to reproduce the dose values measured in the cylindrical water phantoms as specified in Results Section;
(iii) ± 0.5% for f(d,r) factors due to the accuracy of the fits obtained by Eq. (6) (as specified in Results Section) to reproduce the ratios $S_{t}/S\prime_{t}$ measured as a function of the cylindrical water phantom displacement d;
(iv) ± 1.0% due to the inverse square low approximation used in Eq. (2);
(v) ± 1.0% due to the determination of the patient radiological thickness, w', obtained while considering the CT number calibration reproducibility;
(vi) ± 1.0% due to the equivalent square field determination obtained when considering the Sterling approximation;^(^ ^19^ ^,^ ^20^ ^)^
(vii) ± 2.0% due to the linac output factor variability accepted in our center;
(viii) ± 2.0% due the long term EPID calibration stability;^(^ ^21^ ^)^
(ix) ± 2.0% due to the uncertainty of the DPIs alignment versus the DRRs.

Propagating these uncertainties in quadrature, an uncertainty of ± 4.4% (2 SD) was obtained. Because the results of the proposed *in vivo* dosimetry method are reported in terms of ratio between the *in vivo* reconstructed dose ($D_{m}$) and the predicted dose ($D_{m,\text{TPS}}$ computed by the TPS), the TPS calculation uncertainty has to be accounted for in the tolerance level determination. The uncertainty in terms of (2 SD) for the $D_{m,\text{TPS}}$ can be assumed equal to ± 3% ^(^ ^17^ ^)^ in homogeneous tissue regions. Therefore, a tolerance level of ± 5% can be estimated for the ratios between $D_{m}$ and $D_{m,\text{TPS}}$ propagating in quadrature the *in vivo* reconstructed and TPS calculated dose uncertainties. When R or average dose value along the dose profile was beyond the tolerance level, clinical actions were performed to investigate the presence of possible errors in patient setup, machine settings or TPS calculations.

The uncertainty of Eq. (4) used to reconstruct a dose profile in homogeneous regions was estimated equal to 4.5% propagating in quadrature the uncertainty of Eq. (2) equal to ± 4.4% (2 SD) with the exponential term uncertainty that was estimated within ± 1.1% considering the maximum variation of mean energies of 6 MV photon beams, (between 1.7 and 2.2 MeV,^(^ ^16^ ^,^ ^21^ ^)^ that determines a variation in the term $\text{exp}(‐\mu_{\text{en}}\;w\prime_{2}/\rho )$ of 0.5%, and the uncertainty associated to the radiological thickness determination (1.0%). In lung tissue and in regions near the interfaces, further uncertainties should to be introduced to take into account the variation of the scatter contribution with regard to that present in the cylindrical water phantoms where the correlation function was obtained. However, in the present paper, only a preliminary comparison between the *in vivo* reconstructed and TPS‐computed lung doses is reported. Therefore, the complete estimation of the tolerance level of lung dose, that require a specific work, was not performed.

## III. RESULTS

The $S_{t}/D_{m}$ ratios obtained for the 15° and 30° wedges with the two EPIDs were coincident within the measurements reproducibility (± 0.5%). Thus all the $S_{t}/D_{m}$ values were fitted versus w by a polynomial function, F(w), of the second order that was able to reproduce the data well within ± 2.0% (2 SD). Table 1 reports the coefficients of the function F(w), known as the correlation function.

**Table 1:** Coefficients of the correlation function $F(w)=a_{1}w^{2}+a_{2}w+a_{3}$.

$a_{1}$	$a_{2}$	$a_{3}$
$5.819\times 10{‐}^{4}$	$2.880\times 10{‐}^{2}$	$5.480\times 10{‐}^{1}$

The $S_{t}/S{'}_{t}$ ratios showed independence of the cord length, w and the field size (within ± 0.3%), while they were dependent on the cylinder radius, r, and the distances, d, examined.

The $S_{t}/S{'}_{t}$ ratios obtained with the 15° and 30° filters were coincident, within the measurements reproducibility (± 0.5%) for both linacs, and were fitted by linear functions versus the distance, d, by the equation:

(6) $$f(d,r)=f_{0}(r)d+1$$

Table 2 reports the $f_{0}$(r) values obtained for the four cylindrical phantoms.

**Table 2:** $f_{0}$(r) values obtained for the four cylindrical phantoms.

*Phantom Radius (cm)*	*6.0*	*8.3*	*10.7*	*14.5*
$f_{0}$(r)	$0.998\times 10{‐}^{3}$	$1.487\times 10{‐}^{3}$	$2.094\times 10{‐}^{3}$	$2.756\times 10{‐}^{3}$

The accuracy of dose profile reconstruction method was assessed in homogeneous medium by the comparison between the dose profiles measured by the ion chamber in the cylindrical water phantoms and those reconstructed by Eq. (4). An agreement within ± 2% (2SD) was observed independently of the phantom radius and the distance, d, used in the measurements.

**Figure 4:**
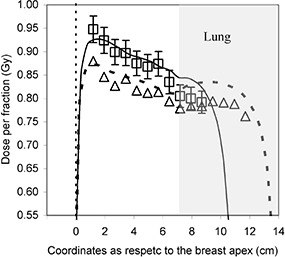
Planned dose profile (continuous line) and average reconstructed dose profile (□); the error bars are representative of the average standard deviation (3%) of the dose points along the profile. Reconstructed dose profile (Δ) and TPS dose profile (dashed line) obtained for the therapy session with a misalignment of 2 cm ((obtained simulating the patient displacement by the TPS). The points at distances of less than 1 cm from the interfaces were not considered for the dose reconstruction.

Figure 4 shows the comparison between TPS computed and *in vivo* reconstructed dose profiles along $\bar{CD}$ for the case reported in Fig. 3. This latter profile was obtained as the average of the *in vivo* dose values measured for the 20 fractions checked for this patient, and the error bars (3%) are representative of one average standard deviation of the dose values obtained for all the therapy sessions. The average dose values agree with the TPS dose profile within 2% and 5% in the breast and in the lung regions, respectively. The dose values reconstructed in the point closer to the breast apex showed a general overestimation. This result seems consistent with the fact that the correlation function does not take into account the reduction of the scatter component present in the breast near its apex. On the contrary, the reconstructed lung doses were underestimated as respect to those computed by TPS. Considering that the correlation function was obtained in homogeneous water phantoms, it does not take into account the reduction in scatter contribution due to the presence of the lung. Therefore, a lung dose overestimation should have been obtained by our method as respect to TPS values. To understand these results, other effects need to be considered such as breathing and organ motion. Moreover, the *in vivo* dosimetry method has to be accurately tested near the interface regions (where the lateral electronic equilibrium is not achieved) and in low density media.

Figure 4 also shows the dose profile reconstructed for the therapy session described in Fig. 3(b) where patient setup misalignment was about 2 cm, and the one computed by TPS simulating the setup misalignment. In this case the reconstructed and the computed dose profiles agree within 3% in breast and 5% in lung. The comparison between the dose profiles reconstructed for the therapy session with a 2 cm misalignment and the one planned for the correct patient setup points out a mean dose variation of −7% inside the PTV. Regarding this dose variation, for the most part, −5%, was due to the different wedge thickness (a 30° filter was used) crossed by the rays which pass through the same breast points when the patient is misaligned. In other words, if the field had been open, even with a patient displacement of 2 cm, dose variation in the PTV would have been small because the rays passing through the PTV would have crossed approximately the same radiological thicknesses planned by the TPS. However, for a wedged field, the relative filter position with respect to the breast changes with patient misalignment; therefore, the filter thicknesses crossed by beam rays to reach the same points in the breast also changes. In a situation of incorrect patient setup, the good agreement between dose profiles reconstructed by Eq. (4) and the ones calculated simulating patient displacement by the TPS points out that if the EPID signal is read aligning the DPI versus the DRR, so reconstructed dose can be considered a realistic estimation of the dose delivered during the therapy session. If the alignment procedure had not been performed, mean dose variation along the profile would have been −17% instead of −7%.

**Figure 5:**
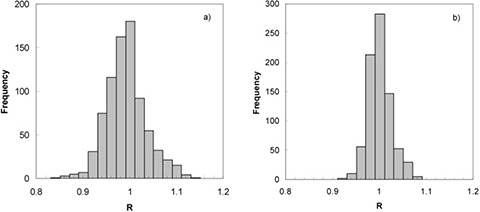
Frequency distribution (a) of the R ratios obtained without taking into account the patient set‐up variations for the 800 DPIs examined; frequency distribution (b) of the R ratios obtained aligning the DPI versus the DRR for the same *in‐vivo* dosimetry checks as shown in Fig. 5(a).

Figure 5(a) shows the frequency distribution of the R ratios obtained without taking into account patient setup variations for the 800 DPIs examined, whilst Fig. 5(b) shows the frequency distribution of the R ratios obtained aligning the DPIs versus the DRRs for the same *in vivo* dosimetry checks shown in Fig. 5(a). In the first case, the R values showed variations with respect to 1 within ± 5% in 72% of the checks, and the mean R value, $\bar{R}$, was 0.98 with a standard deviation of 4.2%. In the second case, the R values showed variations with respect to 1 within ± 5% in 93% of the checks and $\bar{R}$ was 0.99 with a standard deviation of 2.4%.

The average standard deviations, σ, of the patient setup misalignments obtained by the 800 *in vivo* dosimetry checks, found for the breast apex‐central lung and cranio‐caudal directions were 3.5 mm and 5.3 mm, respectively. By comparing DPIs with corresponding DRRs, systematic errors were also determined as the difference between the simulation position and the average position during the treatment. The systematic errors were found randomly distributed with a mean value 1.5 mm towards the breast apex and a standard deviation equal to 3.3 mm.

## IV. DISCUSSION & CONCLUSIONS

As reported by Sabet et al,^(^ ^22^ ^)^ the aSi EPIDs show large deviations from water equivalent behavior due to energy‐dependent response and visible light scattering introduced by the phosphor layer. This behavior can be accounted for by determining the EPID sensitivity as a function of field size, source to detector distance, phantom thickness and phantom distance from the EPID, as suggested by Sabet et al.^(^ ^22^ ^)^ However, for the method applied in the present paper, the EPID was maintained at a fixed distance from the source, and its sensitivity variations with parameters as mentioned above were considered in the correlation function F(w) and with the f(d,r) factors. In fact F(w), defined as the ratio between the EPID transit signal and the dose to water measured in cylindrical water phantoms, was obtained for different field sizes and phantom thicknesses, while the effect of the phantom to EPID distance variations was taken into account by the f(d,r) factors.

In the present work, a two‐dimensional approximation was adopted for the *in vivo* dose reconstruction; therefore, the patient parameters were obtained only by the CT slice that contained the isocenter, and the patients were simulated by cylindrical water phantoms. The dosimetric uncertainty due to the approximation of the breast shape with a cylindrical phantom is difficult to estimate; however, measurements performed in a previous work^(^ ^23^ ^)^ have shown that if the *in vivo* dose point is surrounded by a sphere of homogeneous tissues with a radius equal or greater than 2 cm, the effect of eventual inhomogeneities present in the breast should affect the *in vivo* reconstructed dose less than 1%. On the other hand, if the breast is small it can be approximated with the chest wall that, in first approximation, can be simulated by a cylinder.

F(w) was found independent of beam dimensions, wedge angle and phantoms radius, r, within the experimental uncertainty ± 0.5%. In particular, the correlation functions found in the previous work^(^ ^13^ ^)^ were dependent on r as well. This effect is due to the different detectors used to measure the transit signals. Indeed, the authors^(^ ^9^ ^)^ measured transit signals by an ion chamber positioned in an aluminum cap surrounded by air whilst in the present work, $S_{t}$ was measured by an EPID that reproduced scatter conditions similar to those of an extended phantom. However, the difference on the correlation functions does not affect their accuracy.

In the present paper, the *in vivo* dose determination has been obtained aligning DPIs versus DRRs, and preliminary results are reported concerning the reconstruction of dose profiles perpendicular to the beam central axis and reaching from the apex to the lung, passing through the middle point of the breast.

Dose profiles were reconstructed for 20 therapy sessions of one patient. The average dose values reconstructed along the profile inside the PTV showed an agreement within ± 2% (1 SD) with respect to those calculated by the TPS, excluding the interface region between the breast and the lung. For the therapy session with a patient displacement of about 2 cm, Fig. 4 shows that, when comparing the DPI versus the DRR, a mean underdosage of 7% was observed along the profile inside the PTV in good agreement with the one calculated by the TPS simulating the incorrect patient position. The dose discrepancy of 7% was, for the major part (5%), due to the different wedge thicknesses crossed by rays which pass through the same breast regions. If the *in vivo* dose reconstruction had been made without aligning the $S_{t}$ profiles, an average underdosage of 17% would have been obtained along the dose profile inside the PTV. This difference can be justified considering that, when the patient is misaligned, the radiological thickness crossed by rays passing through a given point in the breast does not correspond to that used to determine the relative dose reconstruction factor C (Eq. 5) and, therefore, the *in vivo* dosimetry loose in accuracy. On the contrary, aligning PDIs versus DRRs, the transit signals produced by rays that cross approximately the same radiological thicknesses in the different therapy sessions are associated with the respective C factors and, in this case, the *in vivo* dosimetry tolerance level can be maintained within ± 5%.

Several papers^(^ ^2^ ^,^ ^26^ ^)^ agree that breast motion from breathing during standard whole breast radiotherapy does not significantly affect dose distribution within breast tissue. This means that the *in vivo* reconstructed dose at the breast midpoint should not be significantly affected by breathing. However, the combined effect of positioning uncertainty and breathing motion can introduce significant deviations between planned and delivered dose distributions to lung. In particular, breathing reduces both the dose gradient inside the lung and the volume of lung tissue receiving high radiation doses, but increases the lung volume receiving lower doses. Our results show an average underestimation of lung dose obtained along the *in vivo* dose profile even if a dose overestimation should have been expected. In fact the correlation function is obtained in homogeneous phantoms and it does not take into account the scatter component reduction due to the presence of a low‐density medium. It is clear that further investigations about the ability of our method to reconstruct the dose in low‐density media and the effect on *in vivo* dosimetry due to breathing have to be performed in order to validate our method for lung dose reconstruction.

When comparing Figs. 5(a) and 5(b), it is evident that the dose discrepancies between reconstructed and calculated doses are greater when patient misalignments are not taken into account for dose reconstruction.

Two studies reported in the literature, where transit dosimetry was applied to tangential breast irradiation, were compared to the present work. The authors^(^ ^13^ ^)^ determined the breast midpoint dose for 100 fields measuring $S_{t}$ on beam central axis using an ion chamber as portal detector. In that center, patient setup was verified before each therapy session by a VPI, and patient misalignment was corrected on‐line before beam delivery. In this manner, 94% of the measured doses were in agreement within ± 5% with the TPS‐computed doses. However, the on‐line patient alignment protocol is time‐consuming and because the dosimetric impact of random setup errors is considered small for the breast tangential technique, it is not adopted by our center.

In a recent work, Nijsten et al.^(^ ^27^ ^)^ determined the dose at a depth of 5 cm on the beam central axis for 2348 fields using a CCD‐based EPID. No corrections were made to take into account patient misalignment, and the action levels were derived from an initial clinical experience as a trade‐off between workload and ability to detect errors, aiming at a 90% rate of dose differences within the action levels. The results show that 85% of the doses measured were within the tolerance level chosen between − 17.5% and + 7.5% of the planned dose. The most frequent errors were caused by an irreproducible set up of the patient with displacements of up to 1.5 cm.

In the present work, by integrating the *in vivo* dosimetry with the EPID geometrical information, the dose at breast midpoint has been determined for 800 fields. The outcome was that 93% of the doses measured were within the ±5% tolerance level. However, if patient misalignments had not been accounted for, the outcome would have been that only 72% of *in vivo* measured doses would have been within the ± 5% tolerance level. This suggests that if the patient setup misalignments are not taken into account, larger tolerance levels have to be adopted as a trade‐off between workload and ability to detect errors. However, tolerance levels higher than 5%–6% can mask other types of errors (such as errors in TPS implementation or in beam calibration), limiting the *in vivo* dosimetry efficacy. Moreover, if the patient misalignments are not taken into account, the dose variations in the PTV are overestimated and no quantitative information can be supplied by the transit dosimetry, thereby reducing its significance. The results of this pilot study indicate that the proposed procedure provides realistic estimates of dose variations in the PTV and also in the case of patient misalignment, and permits adoption of tolerance levels within 5%.

Nijsten et al.^(^ ^28^ ^)^ have recently published a model to predict dose‐volume histogram (DVH) changes due to setup errors in breast treatment using two‐dimensional (2D) transit dosimetry. Their goal is very ambitious. Indeed, with the proposed method, 2D transit dosimetry measurements can directly be translated into DVH parameter changes that are clinically relevant. However the method proposed by Nijsten et al. is not easy to implement because it requires specific competence in software development and 2D EPID calibration. The method reported in the present paper can be implemented without particular effort and permits one to verify the presence of dosimetric variations at the breast midpoint within ± 5% with respect to the TPS dose calculation. With our procedure, 20 minutes are needed to extract the patient parameters from the CT slice, while the *in vivo* dosimetry analysis of each therapy session requires about 10 minutes. To reduce the workload needed for the clinical application of the method, a commercial software could be used for matching the PDI and the DRR images and a dedicated software could be implemented to extract the patient parameters from the CT slice.
